# Cigarette Smoke Containing Acrolein Upregulates EGFR Signaling Contributing to Oral Tumorigenesis In Vitro and In Vivo

**DOI:** 10.3390/cancers13143544

**Published:** 2021-07-15

**Authors:** Han-Hsing Tsou, Hong-Chieh Tsai, Chiao-Ting Chu, Hsiao-Wei Cheng, Chung-Ji Liu, Chien-Hung Lee, Tsung-Yun Liu, Hsiang-Tsui Wang

**Affiliations:** 1 Institute of Food Safety and Health Risk Assessment, National Yang Ming Chiao Tung University, Taipei 112, Taiwan; a12602426@ym.edu.tw (H.-H.T.); tyliu2@ym.edu.tw (T.-Y.L.); 2 Institute of Food Safety and Health Risk Assessment, National Yang-Ming University, Taipei 112, Taiwan; 3 Kim Forest Enterprise Co., Ltd., Taipei 112, Taiwan; 4 Department of Neurosurgery, Linkou Chang Gung Memorial Hospital, Taoyuan 333, Taiwan; Newcomer@cgmh.org.tw; 5 School of Traditional Chinese Medicine, Chang Gung University, Taoyuan 333, Taiwan; 6 Institute of Pharmacology, College of Medicine, National Yang Ming Chiao Tung University, Taipei 112, Taiwan; wangh09@nyu.edu (C.-T.C.); hsiaowei@nycu.edu.tw (H.-W.C.); 7 Institute of Pharmacology, College of Medicine, National Yang-Ming University, Taipei 112, Taiwan; 8 Institute of Oral Biology, School of Dentistry, National Yang-Ming University, Taipei 112, Taiwan; cjliu@ms2.mmh.org.tw; 9 Department of Oral and Maxillofacial Surgery, Mackay Memorial Hospital, Taipei 112, Taiwan; 10 Department of Public Health, College of Health Sciences, Kaohsiung Medical University, Kaohsiung 807, Taiwan; cnhung@kmu.edu.tw; 11 Research Center for Environmental Medicine, Kaohsiung Medical University, Kaohsiung 807, Taiwan

**Keywords:** oral squamous cell carcinoma, cigarette, acrolein, *EGFR* amplification, EGFR signaling pathway, cetuximab

## Abstract

**Simple Summary:**

Oral squamous cell carcinoma (OSCC) is one of the most common smoking-related cancer types in the world. Better understanding of the pathophysiology of OSCC would lead to the development of novel therapeutic options. The epidermal growth factor receptor (EGFR) pathway plays a crucial role in the development of OSCC, and aberrant EGFR expression levels have been associated with smoking. Cigarette smoke contains large amounts of aldehydes such as acrolein, which is a highly reactive environmental toxin. In this study, our results present that acrolein is important in oncogenic transformation through activating the EGFR signaling pathway, contributing to oral carcinogenesis. To the best of our knowledge, this is the first study to provide molecular evidence, showing that cigarette smoke containing acrolein contributes to *EGFR* amplification and activation of downstream signaling in OSCC. Thus, acrolein might be a novel target for early detection and prevention of oral cancer in the future.

**Abstract:**

Oral squamous cell carcinoma (OSCC) accounts for 80–90% of all intraoral malignant neoplasms. The single greatest risk factor for oral cancer is tobacco use, including cigarettes, cigars, chewing tobacco, and snuff. Aberrations of the epidermal growth factor receptor (EGFR) pathway features prominently in oral tumorigenesis and progression. It was shown that cigarette smoking (CS) is associated with worse prognosis in OSCC patients and overexpression of EGFR in tumor tissue. However, the mechanism by which cigarette smoking induced EGFR pathway activation remains to be fully elucidated. Acrolein, an IARC group 2A carcinogen, is a highly reactive aldehyde found in CS. Here we report that acrolein is capable of inducing tumorigenic transformation in normal human oral keratinocytes (NOK). The acrolein-transformed NOK cells showed *EGFR* copy number amplification, increased EGFR expression, and activation of downstream ERK and AKT signaling pathway. No p53 mutations were observed in acrolein-transformed NOK cells. Inhibiting EGFR pathway using an anti-EGFR antibody, cetuximab, inhibits tumor growth. Furthermore, by examining tissue sample from patients, we found an increased *EGFR* copy number was positively associated with acrolein-induced DNA damages in OSCC patients. Taken together, our results indicate that acrolein is important in tumorigenic transformation through amplification of *EGFR* and activating the downstream signaling pathway, contributing to oral carcinogenesis. This is the first study to provide molecular evidence showing that CS containing acrolein contributes to *EGFR* amplification in OSCC.

## 1. Introduction

Head and neck squamous cell carcinoma (HNSCC) is one of the most common smoking-related cancer types around the world. Oral squamous cell carcinoma (OSCC) is the foremost common anatomic site of HNSCC, accounting for roughly 50% of all HNSCC [1]. The predominance of the disease in different parts of the word reflects different forms and extents of expose to these etiological agents. Approximately two-thirds of HNSCC can be ascribed to cigarette smoking, which is classified as group 1 (carcinogenic to humans) by the International Agency for Research on Cancer (IARC) [2,3]. Cigarette-containing cancer-causing agents have harmful effects on upper aerodigestive tract mucosa in a chronic manner, resulting in the accumulation of genetic alterations. In this respect, cigarette smoke truly reaches and damages the entire epithelium covering the upper aerodigestive tract. Unfortunately, with all the advancement in the comprehension of the pathogenesis and recognition of the related risk factors, the 5-year survival rate of OSCC is still 50% [1]. The main challenge to lessen the mortality and morbidity of this disease is to foster techniques to detect and distinguish the OSCC when it is at an early phase, which will enable powerful treatment. Diagnosis of the OSCC is currently dependent on the expert clinical assessment and histological examination of suspicious regions, yet it could be indistinct in hidden sites. In this manner, sensitive and specific biomarkers for OSCC might be useful in screening high-risk patients [4].

Epidermal growth factor receptor (EGFR) is a trans-membrane tyrosine kinase receptor of the ErbB-family, which is accepted to play a crucial role in the development of OSCC [1,5,6,7]. EGFR expression has been related to several downstream pathways, prompting a high tumor proliferation rate, hindrance of apoptosis, improved tumor invasion, and metastasis [8]. EGFR expression and abnormal gene copy number were associated with a poor prognosis of HNSCC patients [9,10,11]; and the anti-EGFR antibody cetuximab has been approved for treating HNSCC [12,13,14,15]. The correlation between smoking and prognosis among OSCC patients has been well established in the literature [16]. Furthermore, it has been shown that EGFR levels correlate with smoking [8,17] and could be utilized to predict survival for OSCC patients [18]. Nonetheless, what substances in CS contribute to EGFR activation remain elusive.

Cigarette smoke (CS) contains in excess of 60 human mutagens, of which polycyclic aromatic hydrocarbons (PAHs) and aldehydes are suspected as the significant CS carcinogens. While PAHs, for example, benzo(a)pyrene, have been demonstrated to be human carcinogens [19], the amount of PAHs in CS is relative minute. Conversely, CS contains relatively large amounts of aldehydes such as acrolein (Acr) (140–550 μg/cigarette), at levels multiple times higher than a known carcinogen, benzo(a)pyrene (5 ng/cigarette) [20,21,22]. Acrolein (2-propenal), the most reactive α,β-unsaturated aldehydes, is a highly mutagenic and highly oxidizing environmental toxin [23]. IARC working groups have re-examined acrolein as probably carcinogenic to humans (Group 2A) [24]. Our previous results have shown that acrolein can damage DNA [25,26,27] resulting in mutations and lead to cancer development [27,28,29]. Furthermore, our previous studies have shown that acrolein contributes to the synergistic potential of CS- and betel quid-induced OSCC [30]. However, the underlying mechanism by which acrolein induces oral tumorigenesis is unclear. In this study, we investigated the effect of acrolein in tumorigenic transformation using normal human oral keratinocytes (NOK) and xenograft tumorigenesis mice models. In addition, EGFR expression and downstream signaling pathway were investigated and the anti-EGFR antibody, cetuximab, was used to verify in acrolein-transformed NOK clones.

## 2. Materials and Methods

### 2.1. Cell Culture and Acrolein Treatment

Human normal oral keratinocyte (NOK) was kindly gifted by Dr. Kuo-Wei Chang at the Institute of Oral Biology, School of Dentistry, National Yang Ming Chiao Tung University, Taipei, Taiwan, and was authenticated [31]. NOK cells were grown in keratinocyte-SFM (KFSM, 1X, Thermo Fisher Scientific, Waltham, MA, USA) medium supplemented with human recombinant 0.2 ng/mL EGF 1–53 and 25 µg/mL bovine pituitary extract Thermo Fisher Scientific, Waltham, MA, USA). Acrolein (Acr) stock solution (Sigma–Aldrich, St. Louis, MO, USA) was freshly prepared before use. Cells at 70% confluency were washed with PBS buffer (Thermo Fisher Scientific, Waltham, MA, USA) and treated with acrolein (7.5 μM) in complete culture medium for 1 month at 37 °C in the dark and acrolein-containing medium was changed every other days.

### 2.2. Cell Viability and Cell Proliferation Assay

Cell viability was performed using a modified 3-(4,5-dimethylthiazol-2-yl)-2,5-diphenyl tetrazolium (MTT; Sigma, St. Louis, MO, USA) assay [32] and sulforhodamine B (SRB; Sigma, St. Louis, MO, USA) assay [33]. For cell viability assay, cells (5000/well) were seeded in 96-well plates overnight, and treated with different concentrations of acrolein for 24 h. For cell proliferation assay, cells (1000/well) were seeded in 96-well plates overnight, and measured every day for 7 days. These experiments were performed at least three times in triplicate.

### 2.3. Flow Cytometry Analysis of Cell Cycle Phases

The procedure was followed as previously described [32]. Briefly, cells were fixed in ice-cold 70% ethanol (Merck, Darmstadt, Germany) for at least 30 min, washed in PBS, and afterward digested with DNase-free RNase A (50 U/mL) at 37 °C for 30 min. Before flow cytometry analysis, cells were resuspended in 500 μL propidium iodide (PI, 10 μg/mL; Sigma, St. Louis, MO, USA) for DNA staining and cell cycle status was analyzed using a Becton–Dickinson FACScan instrument (BD Biosciences, Franklin Lakes, NJ, USA) and Cell Quest software (BD Biosciences, Franklin Lakes, NJ, USA).

### 2.4. Soft Agar Colony Formation Assay

To evaluate anchorage-independent cell growth, soft agar colony formation assay was performed as described previously [34]. Briefly, a bottom layer was formed with a 3-mL aliquot of 1.2% agar in a culture medium in 6-well plates. Then the top layer containing 10,000 cells/well of parental NOK or Acr-NOK clone #4 was mixed with 3 mL of 0.35% agar in a medium and plated on the solidified bottom agar. Plates were cultured and added with two or three drops of the medium to each dish every other day for 30 days. Colonies were stained with 0.005% crystal violet, photographed, and counted. Experiments were performed at least three independent times in triplicate.

### 2.5. Cell Migration Assay

The cell migration assay was performed in a transwell apparatus with 8-μm pore size membranes (Corning) as described previously [35]. Briefly, parental NOK or NOK Acr-clone 4 (5 × 10^4^/well, 6-well plates) was seeded into the upper chamber and the lower chamber contained growth medium supplemented with 10% FBS. After 24 h incubation, the non-migrating cells in the upper chamber were removed with a cotton swap and migrating cells on transwell filters were then fixed with methanol and stained with crystal violet. The number of cells was be counted in six random fields under a microscope at 200× magnification. These experiments were performed at least three times in triplicate.

### 2.6. Quantitative Real-Time PCR

Total RNA were prepared and subsequent real-time RT-PCR analysis of cDNA was analyzed as described previously [32]. The primers (5′-3′) were CTTCTTAAAGACCATCCAGG and TTTCTGGCAGTTCTCCTCTC for EGFR; and CCGTCTAGAAAAACCTGCC and GCCAAATTCGTTGTCATACC for GAPDH. To calculate the relative RNA expression, GAPDH was used as an internal control for all qRT-PCR reactions and compared with control groups. For the gene copy number, DNA was isolated using a PUREGENE^®^ DNA purification kit followed by real-time RT-PCR analysis as described previously [36]. The primers (5′-3′) were AATAGTTGTGCTTTGGGAAGGA and ATTTCCAACTCCACAGAAGCAT for *EGFR*; and AAAGCCGCTCAACTACATGG and TGCTTTGAATGCGTCCCAGAG for *LINE1*.

### 2.7. Western Blot Analysis

Cell lysates were prepared and analyzed as described previously [37]. Briefly, blots were blocked with 5% non-fat milk and hybridized with primary antibodies overnight at 4 °C. The antibodies against P-EGFR, EGFR, RAS, p-AKT, AKT, P-p44/42 MAPK (Erk1/2) (Thr202/Tyr204), p44/42 MAPK (Erk1/2), Cyclin D1, and GAPDH were purchased from Cell Signaling. The antibody against c-myc was obtained from Santa Cruz Biotechnology. The immunodetection was performed using Enhanced Chemiluminescence (ECL) (Millipore Corporation, Billerica, MA, USA). The original Western blot images can be found at Figures S5 and S6.

### 2.8. Xenograft Mouse Model

Animal studies were performed in accordance with the Institutional Animal Care and Use Committee of National Yang Ming Chiao Tung University and carried out according to the Guidelines for Animal Research of National Yang Ming Chiao Tung University (IACUC#1070208rr). Male Balb-c mice (6 weeks old, 25–30 g weight) were used for in vivo experiments. Acolein-transformed cells, NOK Acr-clone #4 (5 × 10^6^ in 50 μL PBS) were inoculated on Balb/c nude mice with orthotopic injection. The mice were anesthetized by isoflurane (AbbVie, Mettawa, IL, USA) and the mouth was opened and balanced out with handhold tweezer. The 29 G needle syringe was then embedded for 5 mm and injected through the external muscle of lower jaw on the right side of mice. Mice were promptly released and cared with a heat lamp.

Mice were evaluated daily and tumor measurements were performed twice per week. Tumor volumes were calculated using the formula: (length × width^2^)/2. Body weight was also examined twice weekly. Tumor samples were excised after sacrifice. The fresh tumor specimens were cut into 2 pieces; portions from one half were fixed in 4% paraformaldehyde and another half was flash frozen in liquid nitrogen and stored at −80 °C until further use.

### 2.9. Collection of Buccal Cells

The procedure of collection of buccal cells was followed as described previously [30]. Eighteen OSCC patients treated at Mackay Memorial Hospital from February 2016 through August 2018 were enrolled for participation in the study and our study protocol was approved by the Institutional Review Board of Mackay Memorial Hospital. For control participants, 20 healthy subjects without habits of smoking or betel quid chewing were recruited for participation in the study in cooperation with the Department of Public Health, Kaohsiung Medical University and our study protocol was approved by the Institutional Review Board of Kaohsiung Medical University (IRB #KMUH-IRB-20110270). Experiments were conducted in accordance with the Declaration of Helsinki principles.

### 2.10. Slot Blot Assay for Acr-dG Detection

Acr-dG adducts in DNA samples were analyzed based on previously described methods [30,38]. Briefly, buccal DNA (0.25 μg) were loaded onto PVDF membranes using a Bio-Dot SF microfiltration apparatus (Bio-Rad, Hercules, CA, USA) and the membrane was probed with anti-Acr-dG mouse monoclonal antibodies [39] followed by WesternDot™ 625 western blotting kits (Thermo Fisher Scientific, Waltham, MA, USA) in accordance with the manufacturer’s instructions. Relative Acr-dG adducts were calculated by the fluorescence intensity of Acr-dG stained with an anti-Acr-dG antibody normalized to the amount of loaded DNA stained with methylene blue.

### 2.11. Statistical Analyses

Student’s *t*-tests were used to determine statistical significance, and two-tailed *p*-values are shown. A minimum of three independent replicate experiments was performed to justify the use of statistical tests. Pearson correlation analysis was used to analyze the correlation between *EGFR* gene copy number and Acr-dG adducts. All statistical analyses were performed using SPSS software version 20.0.

## 3. Results

### 3.1. Acrolein Increased Cell Proliferation, Anchorage-Independent Activity, and Cell Migration Activity in Normal Human Keratinocytes (NOK)

**Figure 1:**
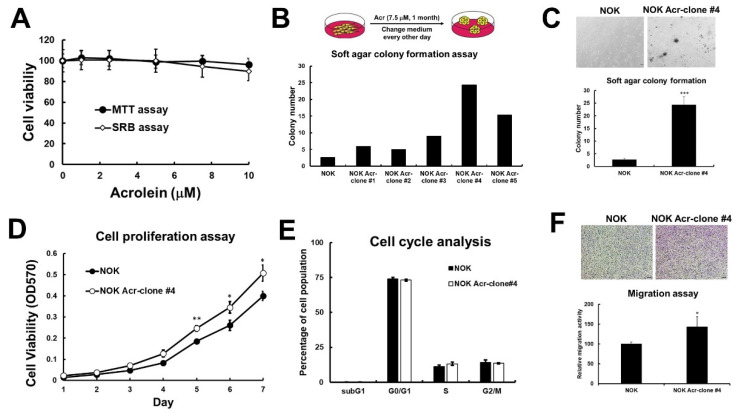
Acrolein induced cell transformation in normal human keratinocytes (NOK). (**A**) Cell viability of NOK under low dose of acrolein (0–10 μM) treatment for 1–3 days was analyzed using MTT assays and SRB assays. (**B**) NOK cells were treated acrolein (Acr, 7.5 μM) for one month and named as Acr-clone #1–5. Anchorage independent cell growth of NOK Acr-clone #1–5 was analyzed using soft agar assays. (**C**) Soft agar anchorage-dependent cell growth of NOK Acr-clone #4 was analyzed using soft agar assays. (**D**) Cell proliferation of NOK Acr-clone #4 was analyzed using MTT assays. (**E**) Cell cycle progression of NOK Acr-clone #4 was analyzed using cell cycle analysis with PI staining. (**F**) Cell migration activity of NOK Acr-clone #4 was analyzed using transwell migration analysis. Data were presented as the mean ± s.d. Student’s *t*-tests were used to determine statistical significance, and two-tailed *p*-values are shown. *** *p* < 0.005, ** *p* < 0.01, * *p* < 0.05 compared with NOK parental cells.

To examine the effect of acrolein in tumorigenic transformation in human oral cells, we cultured normal human keratinocytes (NOK) in medium containing low dose of acrolein (7.5 μM, IC_10_) for one month and selected as NOK Acr-clones, #1–#5 (Figure 1A,B). Anchorage-independent activity of these 5 clones was analyzed using soft agar colony formation assay and the result showed that NOK Acr-clone #4 formed more colony numbers than others (Figure 1B,C). In addition, NOK Acr-clone #4 (doubling time = 35.2 h) has faster cellular proliferation compared to parental cells (doubling time = 42.6 h) (Figure 1D), though no significant difference in cell cycle distribution was observed using cell cycle analysis (Figure 1E). In addition, NOK Acr-clone #4 showed increased migration activity compared with parental cells (Figure 1F) using transwell assay. These results suggest that acrolein increased cell proliferation, anchorage-independent activity, and cell migration activity in vitro.

### 3.2. NOK Acr-Clone #4 Formed Tumors in Xenografts Nude Mice

**Figure 2:**
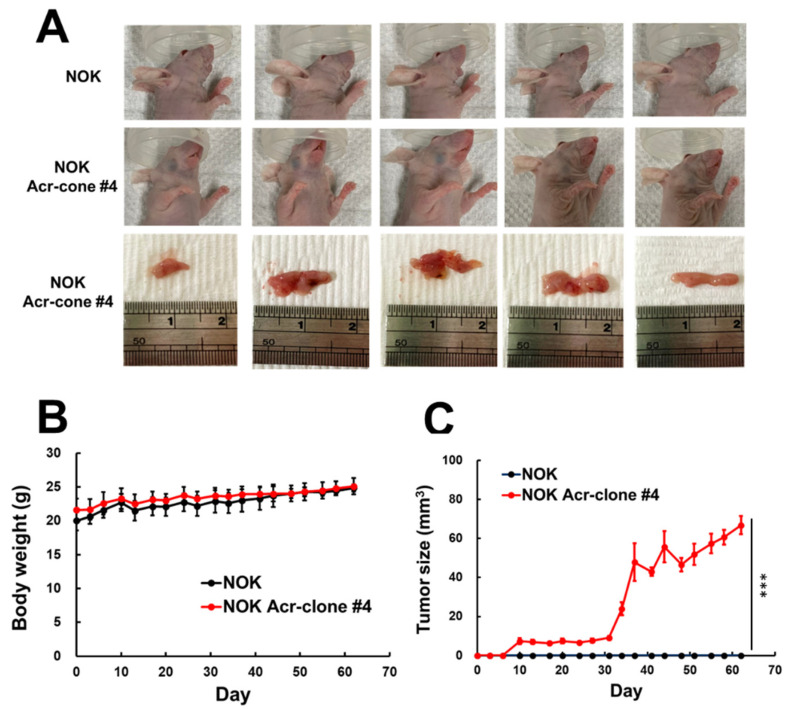
Xenograft mice model of acrolein-transformed clones. (**A**) Overall view of tumors formed by acrolein-transformed NOK cell clone (NOK Acr-clone #4). Tumors in nude mice were seen after injection with acrolein-transformed clone #4, whereas none were seen after orthotopic injection with normal human oral keratinocytes (NOK). (**B**) Tumor growth curves and (**C**) body weight for nude mice of different experimental groups (*n* = 5). Student’s *t*-tests were used to determine statistical significance, and two-tailed *p*-values are shown. *** *p* < 0.005 compared with NOK parental cells.

Our results showed that a long-term low dose of acrolein treatment can transform normal human oral keratinocyte into malignant cells. To further validate its tumorigenic potential, we performed in vivo xenographic tumor implantation. The NOK Acr-clone #4 and parental NOK cells were injected into the buccal area of nude mice and evaluated daily. Nodular neoplasms could be observed at NOK Acr-clone #4 injection site 4 weeks later and continued to grow for 4 more weeks, whereas the parental NOK cells did not form observable tumor up to 8 weeks (Figure 2A–C). These results indicate that acrolein-transformed cells are tumorigenic in vivo, and further supports our hypothesis that acrolein is capable of malignant transforming normal cells into cancer cells.

### 3.3. Acrolein Induced EGFR Amplification and Activated EGFR Signaling Pathway in NOK

**Figure 3:**
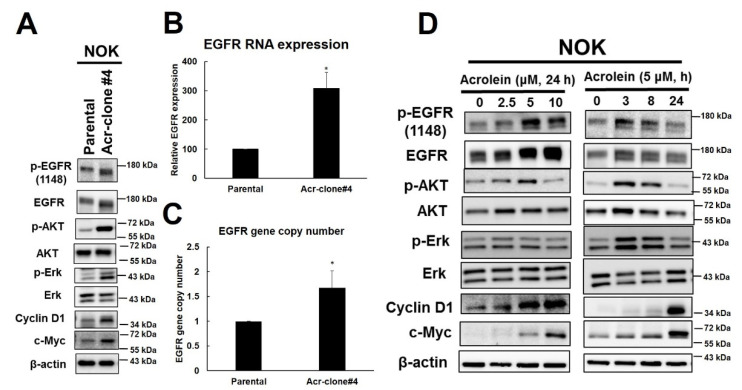
EGFR signaling pathway was activated in acrolein-transformed NOK clone #4 and acrolein treatment-induced activation of EGFR signaling pathway in parental NOK cells. (**A**) Western blot analysis of EGFR pathway (p-EGFR, EGFR p-AKT, AKT, p-ERK, ERK, cyclin D1, c-myc) in acrolein-transformed clone #4 compared to parental NOK cells. (**B**) mRNA expression of EGFR was analyzed in NOK Acr-clone #4 and NOK parental cells using quantitative real-time RT-PCR assays. (**C**) Gene copy number of *EGFR* was analyzed in NOK Acr-clone #4 and NOK parental cells using quantitative real-time PCR. Data was expressed relative to the *LINE1* gene. Student’s *t*-tests were used to determine statistical significance, and two-tailed *p*-values are shown. * p < 0.05 compared with NOK parental cells. (**D**) Western blot analysis of EGFR pathway in NOK cells treated with acrolein in time- and dose-dependent manners.

**Figure 4:**
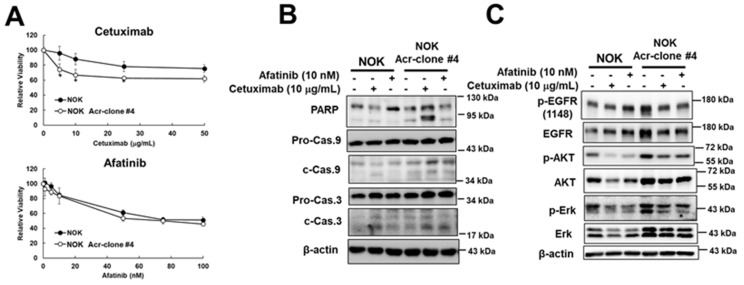
Cetuximab, an anti-EGFR antibody, can induce apoptosis and inhibit EGFR activation in acrolein-transformed NOK clone #4. (**A**) Cytotoxicity of cetuximab (0–50 μg/mL, 48 h) and afatinib (0–100 nM, 48 h) in NOK Acr-clone #4 and NOK parental cells was analyzed using MTT assays. Student’s *t*-tests were used to determine statistical significance, and two-tailed *p*-values are shown. * *p* < 0.05 compared with NOK parental cells. (**B**) Western blot analysis of apoptosis pathway in acrolein-transformed clone #4 and NOK parental cells treated with afatinib (10 nM) or cetuximab (10 μg/mL) for 48 h. (**C**) Western blot analysis of EGFR pathway (p-EGFR, EGFR, p-AKT, AKT, p-ERK, ERK, cyclin D1, c-myc) in NOK Acr-clone #4 and NOK parental cells treated with afatinib (10 nM) or cetuximab (10 μg/mL) for 6 h.

Previous studies have shown that EGFR is highly expressed in oral cancer and correlates with poor prognosis [1,10]. EGFR is a receptor protein tyrosine kinase that plays an important role in regulating the survival, proliferation, and differentiation of epithelial cells as well as tumors of epithelial cell origin [40]. Therefore, we analyzed whether EGFR and downstream signaling were induced in NOK Acr-clone #4 and the results showed that the increase of EGFR and p-EGFR and downstream AKT and ERK pathways as well as increase of cyclin D1 and c-myc were observed (Figure 3A). A similar phenomenon was also observed in NOK Acr-clone #5 (Figure S1). Intriguingly, we found mRNA expression of EGFR was increased in NOK Acr-clone #4 (Figure 3B). In order to investigate whether *EGFR* gene amplification contributes to this phenomenon, we further analyzed the *EGFR* copy number in acrolein-transformed NOK clone cells using quantitative PCR analysis relative to the *LINE1* gene as previously described [7]. The results showed that increased *EGFR* copy number in NOK Acr-clone #4 compared to parental cells (Figure 3C). Furthermore, we also found that short-term treatment of acrolein slightly increased the *EGFR* copy number, activated the EGFR signaling pathway, and increased c-myc in NOK cells in a time- and dose-dependent manner (Figure S2, Figure 3D). In order to further evaluate whether EGFR pathway was involved in acrolein-transformed NOK cells, cetuximab, an anti-EGFR antibody, and afatinib, an EGFR tyrosine kinase inhibitor, were used. The results showed that cetuximab, but not afatinib, was able to cause higher cytotoxicity in NOK Acr-clone #4 compared to parental NOK cells (Figure 4A). Furthermore, we found cetuximab also inhibited soft agar colony formation activity in NOK Acr-clone #4 (Figure S3). Consistently, cetuximab, but not afatinib was able to induce cellular apoptosis in NOK Acr-clone #4 compared to parental NOK cells (Figure 4B). However, both cetuximab and afatinib were able to inhibit EGFR activation and downstream signaling in NOK Acr-clone #4 (Figure 4C). Therefore, these results suggest that acrolein induced cell transformation in oral cells through activation of the EGFR pathway.

### 3.4. EGFR Amplification Was Associated with Acrolein-Induced DNA Damages in OSCC Patients

**Figure 5:**
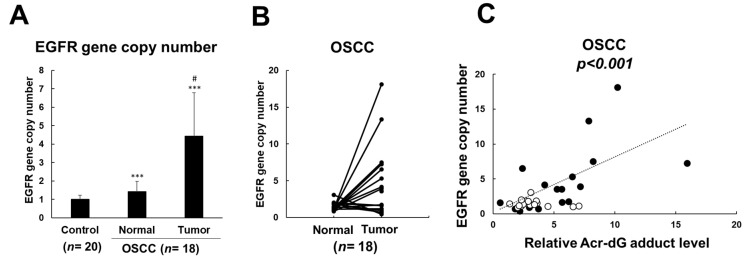
*EGFR* amplification was associated with acrolein-induced DNA damages in OSCC patients. (**A**) Gene copy number of *EGFR* was analyzed in buccal DNA of control subjects and tumor tissues and normal buccal DNA of 18 OSCC patients using quantitative real-time PCR. Data were expressed relative to the *LINE1* gene. Student’s *t*-tests were used to determine statistical significance, and two-tailed *p*-values are shown. *** *p* < 0.005 compared with control subjects, ^#^ *p* < 0.05 compared with normal buccal DNA of OSCC patients. (**B**) Gene copy number of *EGFR* in tumor tissues and counterpart normal buccal DNA of 18 OSCC patients. (**C**) Correlation between *EGFR* gene copy number and relative acrolein levels in 18 OSCC patients was evaluated by Pearson correlation analysis (*p* < 0.001). Relative acrolein-induced DNA (Acr-dG) levels in tumor tissues and counterpart normal buccal DNA of 18 OSCC patients were analyzed using slot blot analysis. Closed circle, tumor samples; open circle, normal buccal DNA.

Our previous studies have shown that increased acrolein-induced DNA damages (Acr-dG adducts) were observed in OSCC patients compared with healthy subjects [30]. In order to confirm whether acrolein induced *EGFR* amplification in clinical samples, we further investigated the *EGFR* copy number in buccal DNA of 18 OSCC patients compared to 20 control subjects (Table 1). The results showed that increased *EGFR* copy number in buccal DNA of OSCC patients compared to those in buccal DNA of control subjects (Figure 5A). Additionally, a substantially increased *EGFR* copy number in tumor tissue DNA compared to those in buccal DNA of OSCC patients was observed (Figure 5A,B). Furthermore, an increased *EGFR* copy number was positively associated with Acr-dG adducts in OSCC patients (Figure 5C, Figure S4). Therefore, these results indicated that acrolein contributes to *EGFR* amplification in OSCC patients.

## 4. Discussion

Cigarette smoking (CS) continues to be the major risk factor for OSCC development [2,3]. Acrolein (Acr), an IARC group 2A carcinogen, is one of the major aldehydes found in CS [24]. Our previous studies have shown that acrolein contributes to the synergistic potential of CS- and betel quid-induced OSCC [30]. However, the underlying mechanism by which acrolein induces oral tumorigenesis is unclear. Abnormalities of epidermal growth factor receptor (EGFR) are related with oral tumorigenesis and progression. More than 80% of invasive HNSCC overexpressed EGFR and excess of EGFR in frequently associated with poor clinical outcome, increased chemoresistance, high recurrence, and low survival rates [1,10,15]. Additionally, EGFR expression remains increased as the tissue progresses from normal mucosa to hyperplasia to dysplasia [15]. Previous studies have shown that EGFR levels correlate with smoking and could be utilized to predict survival for OSCC patients [9,10,11,18]. Here, our results demonstrate that CS-containing acrolein induced cell transformation through amplification of *EGFR* and activation of downstream signaling pathway, and anti-EGFR antibody, cetuximab, was able to halt their growth. Intriguingly, *EGFR* amplification was associated with acrolein-induced DNA damages (Acr-dG adducts) in OSCC patients.

Our previous studies showed that increased Acr-dG adduct levels in OSCC tissue DNA and mutations on *p53* gene were observed in these OSCC patients [30]. In this study, we observed acrolein induced tumorigenic transformation in normal human oral keratinocytes (NOK) (Figure 1) and the acrolein-transformed NOK clone formed tumors (Figure 2). Previous studies have shown that distribution of Acr-dG adducts in the *p53* gene coincides with the *p53* mutational spectrum in lung cancer, suggesting that accumulation of Acr-dG adducts may induce *p53* mutations and contribute to lung carcinogenesis [29]. Therefore, we further analyzed whether *p53* mutations occurred in acrolein-transformed NOK clones and the results showed that no mutation was observed on the exon 5–9 of *p53* gene. Instead, we found the *EGFR* gene copy number was increased and downstream signaling was activated in NOK Acr-clone #4 compared to parental cells (Figure 3A,B). Intriguingly, *EGFR* amplification was observed in tumor tissue DNA compared to those in buccal DNA of OSCC patients (Figure 5B). Additionally, the positive correlation between Acr-dG adducts and the *EGFR* gene copy number was shown in OSCC patients (Figure 5C). Previous studies have shown that EGFR levels were correlated with smoking [9,18]. However, what substances in CS contribute to EGFR activation remain elusive. This study suggested that CS-containing acrolein contributes to *EGFR* amplification of OSCC patients.

Acrolein is the most reactive α,β-unsaturated aldehyde and is abundant in CS, cooking fumes, and automobile exhaust fumes [23]. *EGFR* gene amplification causes numerous types of cancers, including breast cancer, colorectal cancer, lung cancer, and oral cancer [6]. Previous studies have shown that gene amplification results from an aberrant DNA replication and leads up to several hundreds of gene copies integrated either into extrachromosomal double minutes or chromosomal homogeneously staining regions [9]. Our previous studies have shown that acrolein induced mutagenic Acr-dG adducts and inhibits DNA repairs in human cells [26,27,41]. It is reasonable to postulate that acrolein may induce *EGFR* amplification through an aberrant DNA replication. We indeed observed an increased *EGFR* copy number in acrolein-transformed NOK cells compared to parental cells (Figure 3C). However, the exact molecular mechanisms by which acrolein induced *EGFR* amplification need further investigation.

EGFR plays a key role in cell proliferation, cell survival, cell migration, and invasion through downstream signaling pathways including RAS-RAF mitogen-activated protein kinase (MAPK) and PI3K-Akt pathways [5]. It has been suggested that *EGFR* amplification and overexpression can predict the treatment benefits of EGFR-targeted drugs [42]. Tyrosine kinase inhibitors (TKIs), one of the most successful EGFR-targeted therapies, block EGFR-mediated signal transduction by competing with ATP binding site of EGFR [43]. On the other hand, EGFR-specific monoclonal antibody, cetuximab, prevents EGFR signaling transduction by interfering with ligand binding, leading to inhibition of EGFR dimerization and autophosphorylation. In addition, cetuximab has been reported to induce EGFR internalization and destruction [43]. In this study, we found increased p-EGFR and downstream AKT and ERK pathways as well as an increase in cyclin D1 and c-myc observed in acrolein-transformed NOK cells (Figure 3A and Figure S1). Additionally, cetuximab, but not afatinib, was able to cause higher cytotoxicity and cellular apoptosis in acrolein-transformed NOK cells compared to parental NOK cells (Figure 4A,B). However, both cetuximab and afatinib were able to inhibit EGFR activation and downstream signaling in acrolein-transformed NOK cells (Figure 4C). This is consistent with current studies showing that the cetuximab has been proven to work for treating HNSCC [12,13,14,15].

One restriction of this study was the small sample size, which in turn may have incited random chance oscillations during effect size assessment. On the other hand, unknown factors could be influencing the *EGFR* gene copy number or Acr-dG measurements. Another restriction is a single approach to analyze the gene copy number without other verification, for example molecular cytogenetic techniques using fluorescence in situ hybridization with *EGFR*-specific probes [2]. However, regardless of this restriction, the study was able to present the positive association between *EGFR* gene copy number and Acr-dG adduct levels in these OSCC patients. Thus, it would be advisable to independently repeat these results to confirm these findings.

## 5. Conclusions

In conclusion, these results suggest that cigarette smoke containing acrolein is important in tumorigenic transformation through activating EGFR signaling pathway contributing to oral carcinogenesis. To the best of our knowledge, this is the first study to provide molecular evidence, showing that cigarette smoke containing acrolein contributes to *EGFR* amplification in OSCC. Thus, acrolein might be a novel target for early detection and prevention of oral cancer in the future.

## Figures and Tables

**Table 1:** Baseline sociodemographic variables of OSCC patients (*n* = 18).

Characteristic	Count (%)
*Age *(*y*)**	
mean ± SD	56.2 ± 11.7
range	42–89
*Sex *(*n *(*%*))**	
Male	17 (94.4)
Female	1 (5.6)
*Cancer location *(*n *(*%*))**	
Tongue	4 (22.2)
Buccal mucosa	4 (22.2)
Gingiva	4 (22.2)
Hard palate	2 (11.1)
Retromolar trigone	2 (11.1)
Oropharynx	2 (11.1)
*Clinical stage *(*n *(*%*))^^^**	
Stage I	2 (11.1)
Stage II	4 (22.2)
Stage III	1 (5.6)
Stage IV	11 (61.1)
*Cigarette smoker *(*n *(*%*))^#^**	*17 (94.4)*
*Alcohol drinker*(*n *(*%*))** ^#^	*16 *(*33.3*)**
*Betel quid chewer *(*n *(*%*))** ^#^	*17 (94.4)*

^^^ Diagnosis case number for site of primary tumor is 18 and the case number of clinical staging is 18. ^#^ Study participants were asked if they had ever smoked cigarette, chewed BQ, and had alcohol on a regular basis (at least once a week). Those who responded “yes” to these questions were classified as tobacco, BQ, and alcohol users.

## Data Availability

Not applicable.

## Supplementary Materials

The following are available online at https://www.mdpi.com/article/10.3390/cancers13143544/s1, Figure S1: EGFR signaling pathway was activated in acrolein-transformed NOK clone #4 and clone #5. Figure S2: Short-term treatment of acrolein slightly increased *EGFR* gene copy number, Figure S3: Cetuximab inhibited soft agar colony formation activity in acrolein-transformed NOK clone #4. Figure S4: Slot blot analysis of acrolein-induced DNA (Acr-dG) adducts in buccal DNA and tumor DNA in OSCC patients, Figure S5: Original Western blot figures and intensity ratio of each band of Figure 3A,D, Figure S6: Original Western blot figures and intensity ratio of each band of Figure 4B,C.
