# Supplementary material for: Cigarette Smoke Containing Acrolein Upregulates EGFR Signaling Contributing to Oral Tumorigenesis In Vitro and In Vivo
Source: Cancers (Basel). 2021 Jul 15;13(14):3544. doi: 10.3390/cancers13143544 (PMC8307191; doi:10.3390/cancers13143544)
Supplement: Supplementary file 1 [file cancers-13-03544-s001.zip › cancers-1257764-supplementary.pdf]

cancers

Supplementary Material: Cigarette Smoke Containing Acrolein Upregulates EGFR Signaling Contributing to Oral Tumorigenesis In Vitro and In Vivo

Han-Hsing Tsou, Hong-Chieh Tsai, Chiao-Ting Chu, Hsiao-Wei Cheng, Chung-Ji Liu, Chien-Hung Lee, Tsung-Yun Liu and Hsiang-Tsui Wang

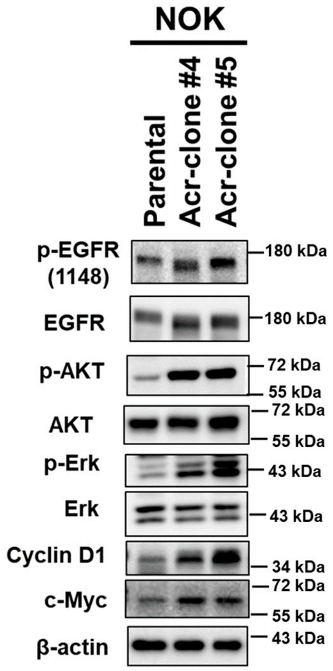

Figure S1. EGFR signaling pathway was activated in acrolein-transformed NOK clone #4 and clone #5. Western blot analysis of EGFR pathway (p-EGFR, EGFR p-AKT, AKT, p-ERK, ERK, cyclin D1, c-myc) in acrolein-transformed clone#4 (shown in **Figure 3A**) and #5 compared to parental NOK cells.

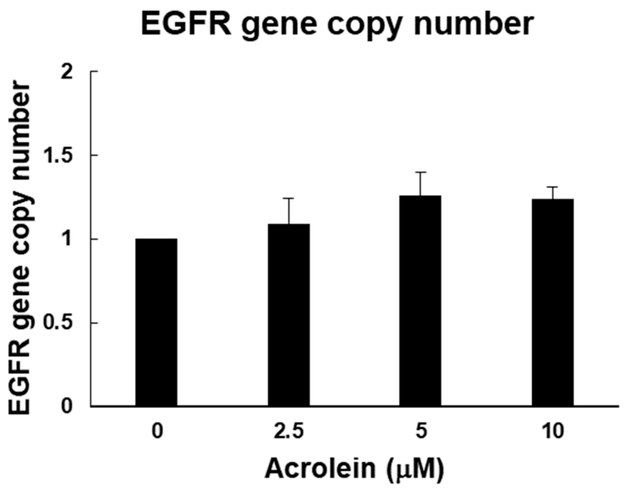

Figure S2. Short term treatment of acrolein slightly increased EGFR gene copy number. Gene copy number of EGFR was analyzed in NOK cells treated with acrolein (0-2.5 M, 24h) using quantitative real-time PCR as described in materials and methods. Data was expressed relative to the LINE1 gene.

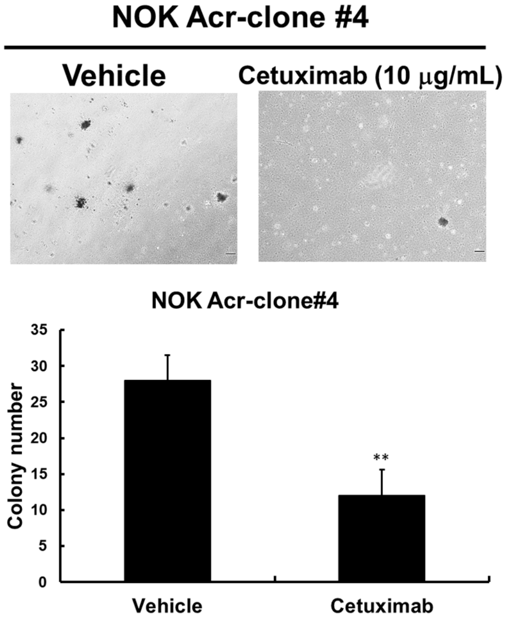

Figure S3. Cetuximab inhibited soft agar colony formation activity in acrolein-transformed NOK clone #4. NOK Acr-clone #4 was treated with PBS (vehicle) or cetuximab (10 g/mL) for 48 h and **s**oft agar anchorage dependent cell growth was analyzed as described in Materials and methods. Data were presented as the mean ± s.d. Student's t-tests were used to determine statistical significance, and two-tailed p-values are shown. **P<0.01.

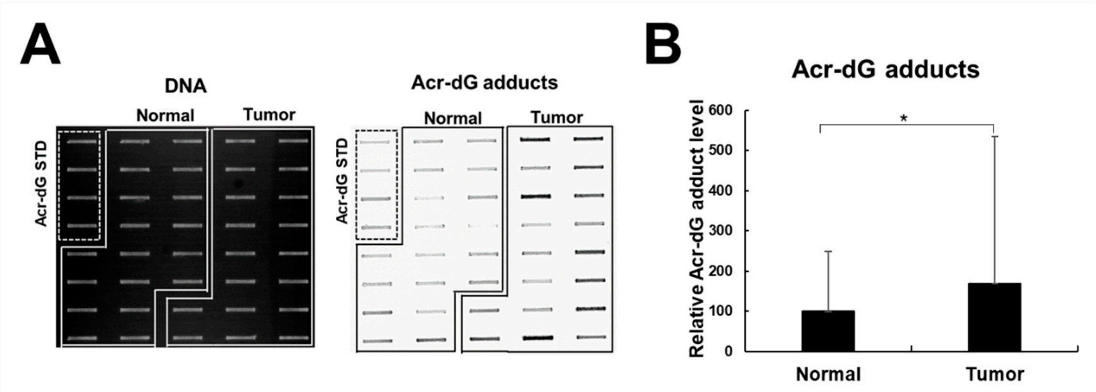

Figure S4. Slot blot analysis of acrolein-induced DNA (Acr-dG) adducts in buccal DNA and tumor DNA in OSCC patients. (A) A representative slot blot result is shown. First, four bands of the first lane, human genomic DNA isolated from cultured NOK cells, were modified with different concentrations of Acr (0, 0.5, 2 and 5 mM) at 37°C for 24 h as Acr-dG standards (Acr-dG STD); the second and third lanes include buccal DNA from OSCC patients; and the fourth and fifth lanes include DNA from tumor DNA of OSCC patients. The left panel shows equivalent amounts of DNA loaded in the membrane that were stained with methylene blue; and the right panel shows fluorescence development and the band intensity was quantified with a UVP image analyzer. (B) Relative Acr-dG levels were calculated by the fluorescence intensity of Acr-dG stained with an anti-Acr-dG antibody normalized to the amount of loaded DNA stained with methylene blue. Relative Acr-dG adduct levels in buccal DNA and tumor DNA of OSCC patients (N=18) as detected by the slot blot analysis described above. Bar graphs of data were collected from 3 independent slot blot experiments. Data were presented as the mean ± s.d. Student's t-tests were used to determine statistical significance, and two-tailed p-values are shown. *p<0.05.

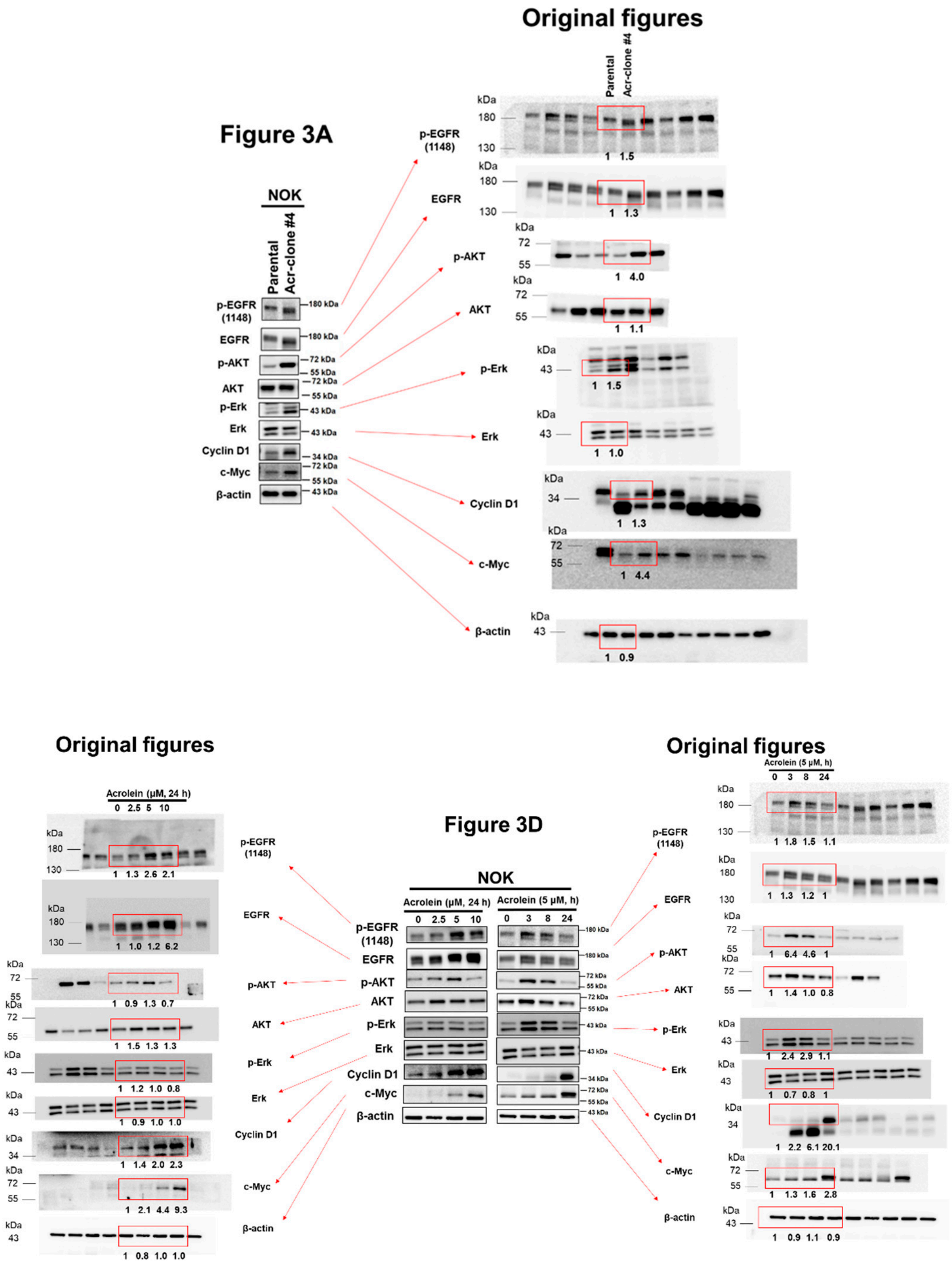

Figure S5. Original Western blot figures and intensity ratio of each band of Figure 3A and 3D.

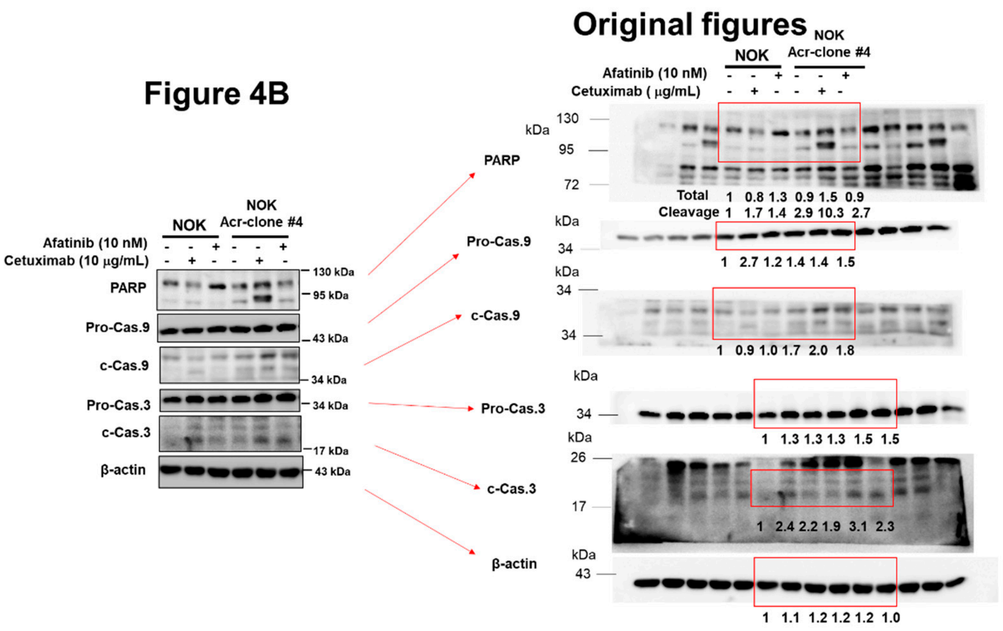

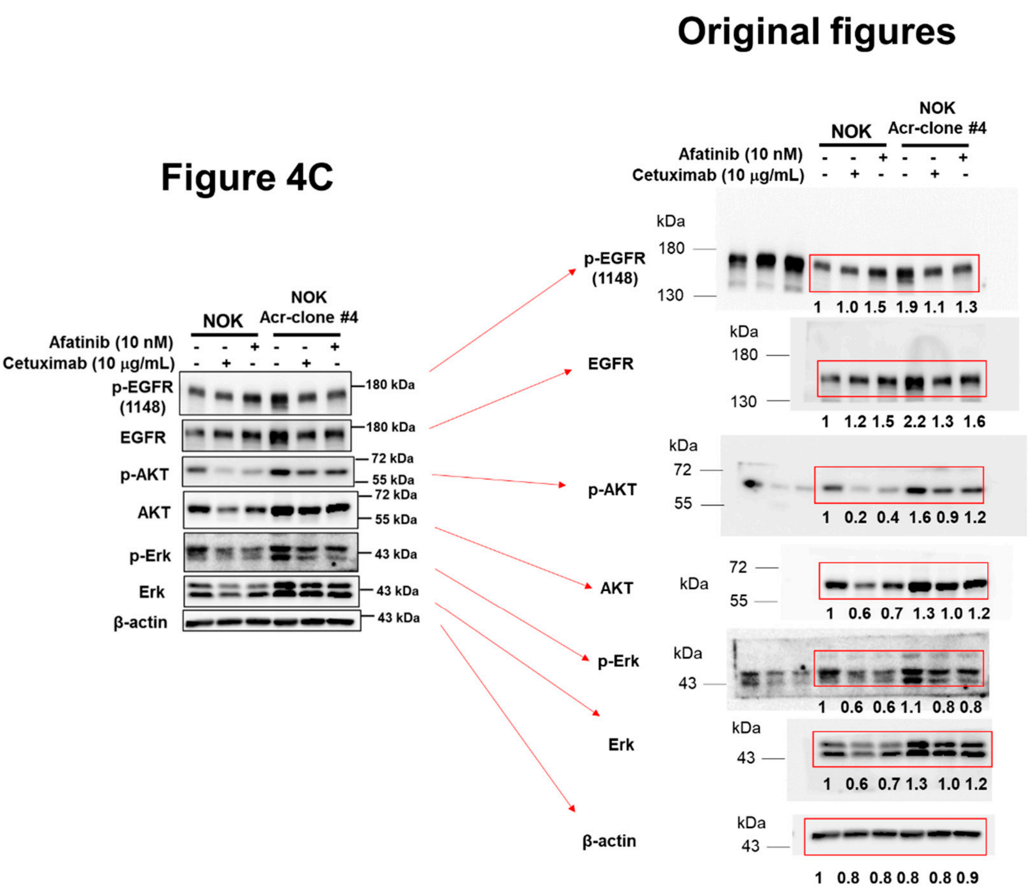

Figure S6. Original Western blot figures and intensity ratio of each band of Figure 4B and 4C.
